# Broad-Spectrum Antimicrobial Epiphytic and Endophytic Fungi from Marine Organisms: Isolation, Bioassay and Taxonomy

**DOI:** 10.3390/md7020097

**Published:** 2009-04-17

**Authors:** Yi Zhang, Jun Mu, Yan Feng, Yue Kang, Jia Zhang, Peng-Juan Gu, Yu Wang, Li-Fang Ma, Yan-Hua Zhu

**Affiliations:** Department of Biotechnology, School of Environmental and Chemical Engineering, Dalian Jiaotong University, Dalian 116028, P.R. China; E-Mails: mujun@djtu.edu.cn; fengyan@djtu.edu.cn; kangyue0823@163.com; zhangjia88624@163.com; juice_juan@yeah.net; wangyu4126@126.com; malifang2009@126.com; xiao_zhu9@yahoo.com.cn

**Keywords:** Epi- and endophytic, marine derived fungi, antimicrobial activity, taxonomy

## Abstract

In the search for new marine derived antibiotics, 43 epi- and endophytic fungal strains were isolated from the surface or the inner tissue of different marine plants and invertebrates. Through preliminary and secondary screening, 10 of them were found to be able to produce broad-spectrum antimicrobial metabolites. By morphological and molecular biological methods, three active strains were characterized to be *Penicillium glabrum*, *Fusarium oxysporum*, and *Alternaria alternata*.

## 1. Introduction

Since the discovery and application of penicillin, antibiotics have saved billions of lives and played an important role in human history. Unexpectedly, many pathogenic microorganisms, e.g. methicillin-resistant *Staphylococcus aureus* (MRSA) and vancomycin-resistant *Enterococcus faecium* (VREF), have developed resistance towards current antibiotics and this trend has become more and more serious [1]. Additionally, some almost vanished pathogens such as *Mycobacterium tuberculosis* have revived and threaten the public health again; meanwhile some new emerging infectious diseases, e.g. cryptococcal meningitis and toxoplasmosis, have also become prevalent [1,2]. All these new problems call for more novel antibiotics.

Encouraged by the idea of “Drugs from the Sea”, the chemists have identified lots of bioactive compounds with novel structures from the rich marine bio-resource in the recent fifty years [3–5]. Among them, marine derive fungi have contributed an important proportion. Many marine fungal strains were isolated, screened, and reported to produce novel antimicrobial compounds belonging to the alkaloids, macrolides, terpenoids, peptide derivatives and other structure types [6–8]. These marine fungal derived compounds have provided us with new choices with which to fight infectious diseases. For example, the marine fungus *Pestalotia* sp. isolated from the surface of the brown alga *Rosenvingea* sp. was able to produce a new chlorinated benzophenone compound pestalone, which showed potent antibiotic activity against MRSA (MIC=37 ng/mL) and VREF (MIC=78 ng/mL), revealing its potential as new antibiotic. It was also interesting that this fungus produced pestalone only when it was co-cultured with a marine bacterium strain CNJ-328, suggesting that the production of this antibiotic was stimulated by bacterial competition [9]. This example may explain why these marine fungi produce antimicrobial secondary metabolites. These antibiotics may be used as chemical defense weapons against other microorganism in the marine environment, for the producers themselves or even for their host.

Epiphytic and endophytic fungi live on the surface and in the inner tissues or even in the cell of their hosts, respectively. Current studies indicate that complex interactions existe between the host and their epi/endophytic fungi, e.g. the host provides organic nutrition and epi/endophytes act as chemical guards [6,10], so compared with free-living marine fungi, the epi/endophytic marine fungi have drawn more interest from natural product chemists in the search for novel antimicrobial or other active compounds. By Bugni and Ireland’s statistics, among the 272 new compounds reported from marine derived fungi till 2002, over 85% were produced by epi/endophytes isolated from plants, invertebrates, and woody substrates [6].

Until now, most of the isolation and screening of marine epi- and endophytic fungi reported was from tropical sea areas; some temperate sea areas with high biodiversity were relatively ignored. Dalian is located on the tip of the Liaodong Peninsula, between China’s inner sea Bohai Sea and the Yellow Sea. Its marine ecological environment is influenced by the Yellow Sea Warm Current, Western Liaoning Coastal Current, Southern Liaoning Coastal Current, and the freshwater from some rivers, which has created diversified habitats suitable for marine plants, invertebrates, and microorganisms. As a less investigated zone, the Dalian sea area probably embodies a rich source of marine derived fungi capable of producing novel antimicrobial compounds. The purpose of this study was to search for antibiotic-producing epi- and endophytic fungal strains from the local marine habitats. In this paper, we report the isolation, bioassay, and taxonomy of broad-spectrum antimicrobial epi- and endophytic fungal strains from the local seaweeds, sea grass, sponge, and other invertebrates.

## 2. Results and Discussion

### 2.1. Sample Collection and Strains Isolation

In the intertidal zone of the Fujiazhuang coastline (121° 36′ 13.82″ E, 38° 48′ 36.66″ N) of Dalian, four species of red alga, two species of brown alga, two species of green alga, one species of higher plant (sea grass), and three species of invertebrates were collected, as shown in Table 1. Although this sampling was carried out in October when the water temperature was lower than in the blooming summer, the result still reflected the biodiversity of this sea area. It was worth noting that the rich *Hymeniacidon perlevvis* sponge resource found in the Dalian sea area possibly contains a large amount of marine fungi for drug discovery. The fungal isolation from these organisms resulted in 43 strains, including 23 epiphytic isolates and 20 endophytic ones. Comparing the origins, the sponge *Hymeniacidon perlevvis*, the brown alga *Sargassum thunbergii*, and the red alga *Gracilaria lemaneiformis* yielded many more cultivable isolates than other substrates.

### 2.2. Preliminary antimicrobial assay

**Figure 1:**
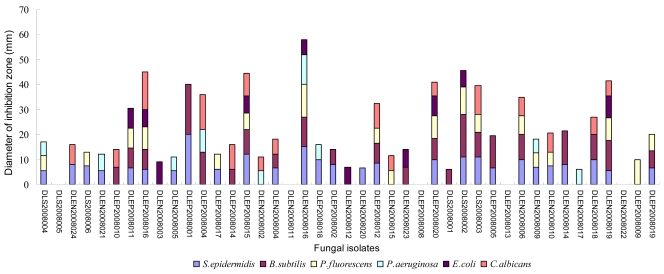
The antimicrobial activities of the fungal isolates against six test microorganisms in the preliminary assay.

The disks cut from the 7-day PSA (potato sucrose agar) plate cultures of the 43 isolates were screened for their antimicrobial activity against six test organisms spread on specific medium plates. The result showed that 88.4% of the epi- and endophytes possessed inhibitory activities against one or more test organisms (Figure 1). 79.1% inhibited Gram positive bacteria (*Staphylococcus epidermidis* and/or *Bacillus subtilis*), 62.8% inhibited Gram negative bacteria (*Pseudomonas fluorescens*, *P. aeruginosa*, and/or *Escherichia coli*), and 48.9% inhibited both G^+^ and G^−^ bacteria. It was also found that 37.2% of them could inhibit the growth of fungus *Candida albicans* besides test bacteria. Ten isolates (23.3%) were able to inhibit no less than four kinds of test organisms.

**Figure 2:**
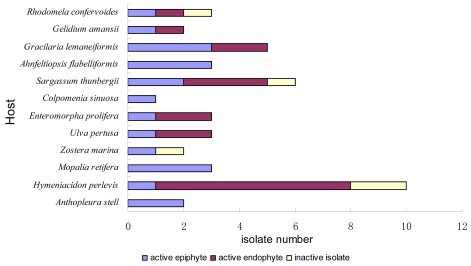
The host origins of antimicrobial fungal isolates.

As for their origins (Figure 2), the results revealed the common existence of antimicrobial epi/endophytic fungi from different hosts, including seaweeds belonging to three phyla, marine vascular plant, and three types of invertebrates, including coelenterate, mollusc, and sponge. The broad existence of antimicrobial epi-/endophytes, was probably originated from nutritional competition between epi-/endophytes and other marine organisms, or was due to their participation in the hosts’ chemical defensive mechanisms, as proposed by Bugni *et al* [6]. Compared with other hosts, the red alga *Gracilaria lemaneiformis*, the brown alga *Sargassum thunbergii*, and the sponge *Hymeniacidon perlevis* provided more activite fungal isolates.

**Figure 3:**
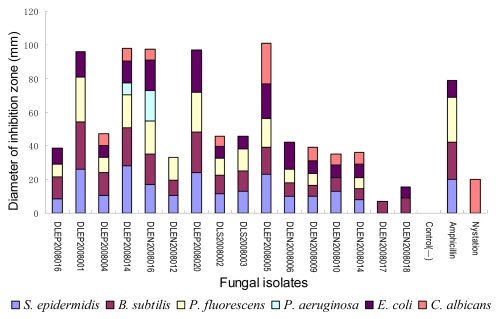
The antimicrobial activity of 16 isolates in the secondary assay (paper disk agar diffusion method). Positive control: ampicillin (dose: 60 μg/mL) for antibacterial test, nystatin (dose: 200 μg/mL) for antifungal test; negative and blank control: methanol.

However, antimicrobial substances secreted by microorganisms could be small molecular secondary metabolites, i.e. antibiotics, or large molecules such as polysaccharides, peptides, enzymes and so on. To determine the ability of the above active strains as antibiotic-producers, sixteen isolates (listed in Figure 3), with relatively broader antimicrobial spectrum or stronger activities shown in preliminary assay, were selected for secondary assay.

### 2.3. Secondary antimicrobial assay

After 7 days’ fermentation in PSB (potato sucrose broth) on a shaker, the ethyl acetate-methanol extracts of 16 selected active strains were re-screened of their antimicrobial activity using a paper disc agar diffusion method. The dose of extract on each paper disc (50 μL from 2.5 mL in total) was equal to the amount of extractable substances in 1 mL of ferment liquid.

As shown in Figure 3, the 16 active isolates were indeed able to produce some antimicrobial organic substances under the condition of aerobic liquid fermentation. Among them, 15 isolates’ activity spectrum covered both G^+^ and G^−^ bacteria, 13 isolates could produce antibiotics inhibiting no less than four kinds of test organisms, and eight isolates could inhibit both bacteria and fungi. It was interesting that most of the antibacterial fungi could inhibited both of the two G^+^ bacteria and no less than two kinds of G^−^ bacteria, revealing the possible existence of some antibiotics with broad-spectrum antibacterial mechanism or/and diverse antibiotics with different specific mechanisms. The phenomenon that all the antifungal isolate possessed anti-G^+^ and G^−^ activity also supported the above deduction.

It was noteworthy that five isolates not only exhibited broad spectrum activity but also were potent enough to compare with the positive controls ampicillin and nystatin. They were isolate DLEP2008001 from the red alga *Ahnfeltiopsis flabelliformis*, isolate DLEP2008014 and DLEN2008016 from the brown alga *Sargassum thunbergii*, isolate DLEP2008020 from the sea grass *Zostera marina*, and isolate DLEP2008005 from the sponge *Hymeniacidon perlevvis*. The diameters of their inhibition zones against specific test organisms were close to or even larger than those of the positive controls; furthermore, isolates DLEP2008014, DLEN2008016, and DLEP2008005 showed broader antimicrobial spectra than ampicillin and nystatin against the test organisms.

As is known, in hospitals *Pseudomonas aeruginosa* is an important prevalent pathogen that often causes infections after surgeries and other infections and many strains of this bacterium have developed drug resistance towards many antibiotics, e.g., penicillins, some cephalosporins and cotrimoxazole [11]. Fungal isolate DLEP2008014 and DLEN2008016’ crude extracts, especially the latter, showed considerable inhibitory activity against *P. aeruginosa*, suggesting their potential use as new anti-*P. aeruginosa* antibiotic-producers. Yeast *Candida albicans* could cause human genitalia infections, e.g., vaginitis, and oral infection of infants and AIDS patients [12,13]. Isolate DLEP2008005’s products inhibited *C. albicans* remarkably and were worth investigating for its effective principals. Some *Staphylococcus epidermidis* and *P. fluorescens* isolates were also pathogens of some prostatitis, septicaemia, and infectious shock [14–16], therefore the five isolates’ unignorable activity against these two test bacteria were also valuable for further study.

### 2.4. Molecular and Morphological Taxonomy

To determine the taxonomy of the three isolates with the broadest antimicrobial profiles, the isolates DLEP2008014, DLEN2008016 and DLEP2008005 their ITS1-5.8S-ITS2 ribosomal DNA sequence information and morphological characteristics were investigated.

Their ITS1-5.8S-ITS2 rDNA sequences were amplified by PCR and the lengths were 501 bp for the isolate DLEP2008014, 515 bp for the isolate DLEN2008016, and 463 bp for the isolate DLEP2008005, respectively. The sequence data had been submitted to and deposited at GenBank (accession numbers: FJ618522, FJ618520, and FJ618521, sequently).

**Figure 7:**
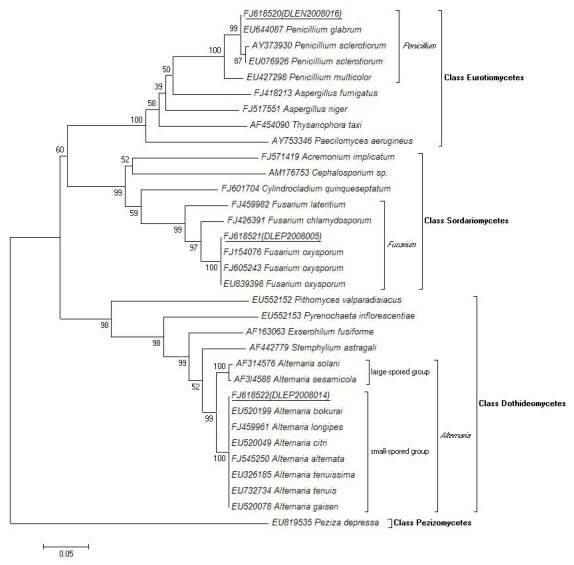
Phylogenetic tree of ITS1-5.8S-ITS2 rDNA sequences of the three isolates, DLEP2008014, DLEN2008016, DLEP2008005, compared with sequences obtained from public databases. NJ-tree was constructed using ITS1-5.8S-ITS2 rDNA sequences. Bootstrap values were calculated using 1,000 replications and *Peziza depressa* was used as out-group.

A BLASTN search of the ITS (internal transcribed spacer) of the isolate DLEP2008014 revealed that it was highly similar to several small-spored species of genus *Alternaria* in GenBank, including *A. alternata* (identity: 100%), *A. longipes* (100%), *A. tenuis* (100%), *A. tenuissima* (100%), *A. bokurai* (100%), *A. gaisen* (100%), and *A. citri* (99%). In a phylogenic tree constructed by NJ algorithm (Figure 7), the isolate DLEP2008014 was also placed on the same clade with the above species (degree of confidence: 100%) while clearly branched from the large-spored group of this genus (supported by 1000 bootstrap replicates).

To identify this isolate with extremely similar ITS sequence to several *Alternaria* species, its colonial and microscopic morphology were studied. After 7 days of cultivation on 1/4 PCA plates at 25°C under natural illumination, the colonies were embossed, floccose, greenblack in center and white on margins on the top, and brown on the reverse side, reaching the diameter of 46–52 mm. After 5 day’s cultivation on PDA plates at 25°C in darkness, the colonies were embossed, light olive-grey, and floccose on the top, and brown on the reverse side, reaching the diameter of 45–55 mm. Its conidiophores were extended by sympodial branching, pale to brown, multi-septate, 17–40 × 3.5–4.5 μm. The conidia were solitary or in chain, brown, subclavate or ovoid or ellipsoid, smooth-walled or with tiny thorns, transversely 3–10-septate and longitudinally or obliquely 0–3-septate, with cylindric or tapered short rostra, 8–35 × 2.5–4.5 μm. Its remarkable characteristics included branching conidia chains with 1–5 conidia on long side branch, short rostra of the conidia, non-incrassate septum, which were in accordance with the characteristics of *Alternaria alternata* (Fr.) Keissl [17]. So, on the basis of molecular analysis and morphology, the isolate DLEP2008014 was identified as *A. alternata* (Fr.) Keissl.

As was known, although the ITS1-5.8S-ITS2 sequences were commonly used as a taxonomic tool on the level of species within a genus in fungal identification for their fairly high divergency [6], it was limited when distinguishing closely related species that had identical or nearly identical ITS sequences, e.g., the small-spored group of Genus *Alternaria* [18,19]. IGS region with the biggest variation might be used as a useful tool to resolve this question, however by now its application was still limited by the poorly accumulated sequence data in open databases such as GenBank. Thus, to identify the species of Genus *Alternaria*, the ITS were recommended to distinguish small-spored group and large-spored group, while for exact identification to species level, the classic morphology was now still the most credible method.

The BLASTN result of the ITS of the isolate DLEN2008016 showed high similarity to *Penicillium glabrum* (99%) and *Penicillium sclerotiorum* (98%). The phylogenic tree also exhibited similar genetic relationship. After 7 days of cultivation on MEA plate at 25°C in darkness, its colonies were velutinous and a little rugous, consisted of white to yellowish mycelia covered by grey-green sporulating structures, didn’t produce exudate, and attained a diameter of 22–30 mm. On the reverse side, the colonies were yellowish brown. The conidiophores were smooth, 58–120 × 3.0–3.5 μm, inflated in the end (4–4.5 μm), monoverticillate, terminating in a whorl of 3–7 flask-shaped phialides (5.8–8.0 × 2.5–3.5 μm) with short necks. The conidia were globose to subglobose, smooth-walled, 2–3 μm. According to the above characteristics, the isolate DLEN2008016 was identified as *Penicillium glabrum* (Wehmer) Westling [20].

As for isolate DLEP2008005, its ITS BLASTN result only showed 100% identity with a lot of isolates of *Fusarium oxysporum*. And the phylogenic analysis also showed its clear differentiation with other close species in the genus of *Fusarium*, firmly supporting the BLASTN result. The conclusion of molecular taxonomy was also supported by the following morphology investigation.

After five days in darkness and three days under natural illumination of cultivation on PDA plate at 25°C, its colonies were floccose, white to purplish, and attained a diameter of 22–30 mm. On the reverse side, the colonies were grey purple. The microconidia were oval-ellipsoid, straight or a little curved, generally abundantly, borne in groups on the top of phialides, mostly non-septate, 3.5–7.0 × 1.7–2.5 μm. Simple phialides were short, flask-shaped to cylindrical, 9.6–25 × 2.1–3.0 μm, arising solitarily and laterally on the hyphae or from its short branches. Macroconidia were sparse, falcate, 3-(5)-septate, 32.2–40 × 3.5–4.5 μm, with a beak shaped apex and a small basal cell. Chlamydospores were globose to subglobose, generally solitary, occasionally terminal or intercalary in pairs or in chains, hyaline, smooth-walled, 6.0–9.0 μm. According to the above morphological characteristics and the ITS information, the isolate DLEP2008005 was identified as *Fusarium oxysporum* Schltdl [21].

Therefore, by molecular and morphological methods, the above three active fungal strains were identified as *Alternaria alternata, Penicillium glabrum*, and *Fusarium oxysporum*. The phylogenetic analysis showed clear homology of the three isolates with respective reference species and their evolutionary relationships with other species in the same genus, family, or class (Figure 7). It was noticed that the three antimicrobial Ascomycota isolates belonged to three different classes, Eurotiomycetes, Sordariomycetes, and Dothideomycetes, respectively.

Until now, *Alternaria* spp. were not commonly reported as producers of antimicrobial antibiotics [22–23], the related reports of marine derived isolates were even more scarcer [24], so the broad antimicrobial profile of *A. alternata* isolate DLEP2008014 is probably of value for the discovery of new antibiotics.

As is known, terrestrial and marine *Penicillium* spp. are important antibiotic-producers [6–7, 25–26], so it was not difficult to understand that the endophytic *P. glabrum* isolate DLEN2008016 of alga *Sargassum thunbergii* was able to produce broad-spectrum antimicrobial substances, which maybe hinted at some chemical defense function. This *P. glabrum* isolate of new origin could also possibly be a producer of new antibiotics.

Terrestrial *Fusarium avenaceum*, *F. bostrycoides*, *F. solani*, *F. equiseti, F. oxysporum* and some unidentified *Fusarium* spp. were reported to produce structurally diversified antimicrobial compounds [27–35], and three marine derived *Fusarium* spp. were also been reported to produce the novel antibacterial cyclodepsipeptide enniatin B [36], the sesterterpene neomangicol B [37], and the antifungal compound fusarielin E [38], which revealed the potential of fungi of genus *Fusarium* as producers of abundant novel antibiotics, so the *F. oxysporum* isolate DLEP2008005 derived from sponge was also expected to be a good antibiotic-producer candidate.

## 3. Conclusions

In total 43 epi- and endophytic fungi were isolated and cultured from each sample of the eight species of alga, one species of higher plant (sea grass), and three species of invertebrates (sponge, coelenterate, and mollusk) collected at intertidal zone of Dalian, China. The bioassay data showed globally that each host was inhabited by active epi and/or endophytic fungi capable of inhibiting one to six test organisms. This phenomenon was speculated to be derived from the natural need of chemical defense for the epi/endophytes themselves or for their hosts in marine ecosystem.

Compared with the other hosts, the red alga *Gracilaria lemaneiformis*, the brown alga *Sargassum thunbergii*, and the sponge *Hymeniacidon perlevis* provided more culturable epi/endophytic strains and more active ones as well. Furthermore, two strains from *S. thunbergii* and one strain from *H. perlevis* exhibited remarkable inhibitory activity against five or all of the Gram positive *Staphylococcus epidermidis* and *Bacillus subtilis*, the Gram negative *Pseudomonas fluorescens*, *P. aeruginosa*, and *Escherichia coli*, and the yeast *Candida albicans*. Compared with the positive control ampicillin and nystatin, the three strains showed comparable activity and even broader antimicrobial spectrum. By molecular and morphological taxonomy, they were identified as *Alternaria alternata* isolate DLEP2008014, *Penicillium glabrum* isolate DLEN2008016, and *Fusarium oxysporum* isolate DLEP2008005. On count of their broad-spectrum antimicrobial activity and previous related reports, these three marine epi/endophytic fungi were expected to be new potential producers of new antibiotics. Their origin, taxonomy, and bioactivities should be also of some value in promoting our understanding of marine ecosystem.

## 4. Experimental Section

### 4.1. Sample Collection and Strains Isolation

The samples were collected from the intertidal zone of Fujiazhuang coast in Dalian City of Liaoning Province, China, in October of 2008. The fresh and healthy samples were identified *in situ* and put into sterile bottles with seawater of the site to keep fresh in short time and then taken back to laboratory in less than 2 hours. On a clean air bench, the samples were washed with sterile seawater two times, then gently shaken with sterile glass beads in flasks at 60 rpm for 10 min on shakers, and finally washed again with sterile seawater two times to remove temporarily and loosely adhering free-living fungi. After the above process, the samples were transferred onto a PSA plate (containing boiled juice of 200 g potato pieces/L, sucrose 20 g/L, chloramphenicol 0.2 g/L, agar 20 g/L, crude sea salt 20 g/L) and rubbed on the medium surface to inoculate the epiphytic fungi. Subsequently, these samples were put into 75% aqueous ethanol for 10 seconds to kill the residual epiphytes, washed with sterile seawater to remove the ethanol, and dried with sterile tissue paper. Then the samples were cut into pieces using a sterilized knife and transferred onto PSA plate to let the kerfs close to the medium to inoculate endophytic fungi. The plates were incubated at 28°C for 3~7 days to observe the emerging mycelial growth. The colonies were purified using repeated hyphal tipping method until pure epi/endophytic cultures were obtained. To determine the endophytes, the colonies from surface-inoculating plates and tissue pieces-inoculating plates were compared, and only the colonies grew from the kerfs of tissue pieces and not from the surface were identified as true endophytic fungi [39]. The pure strains were inoculated onto agar slants and immersed with sterile liquid paraffin for preservation at 4°C.

### 4.2. Preliminary antimicrobial assay

For antimicrobial assay, *Staphylococcus epidermidis* ATCC 12228 and *Bacillus subtilis* ATCC 6633 were used as examples of Gram positive test bacteria, *Pseudomonas fluorescens* ATCC 13525, *P. aeruginosa* ATCC 9027, and *Escherichia coli* ATCC 11229 were adapted as the Gram negative test bacteria, and *Candida albicans* ATCC 10231 was selected as the test fungus. After incubation for 24–48 h at 37°C, the test organism lawns were washed with 0.85% NaCl sterile solution and diluted to 0.5 MCF to be used as standard inoculum suspension, which was stored at 4°C and used within 7 days. The specific medium for test bacteria or fungi were poured into Petri dishes and inoculated with 100 μL of the suspension. Then the inoculums were distributed by a sterile glass rod (L-shaped) on the surface of the medium. The Mueller Hinton Agar medium was used for cultivation of the test bacteria, and the Sabouraud Dextrose Agar medium was used for cultivation of *C. albicans*.

Disks (5 mm diameter) of the cultures of the fungal isolates (7 days old-grown on PSA plate at 28°C) were cut using a sterile borer and picked up to place on the surface of the above medium seeded with test organisms [40]. The plates were refrigerated at 4°C overnight for complete diffusion of antibiotics, thereafter they were incubated at 37°C for 16 h. The diameter of the inhibition zone was measured and the average of three repeated agar disks was taken to assess the strength of antimicrobial activity.

### 4.3. Secondary antimicrobial assay

Fungal strains selected by preliminary screening were inoculated into 250 mL conical flasks containing 50 mL of PSB broth medium and were shaken on a thermostatic shaker with the rotary speed of 180rpm at 28°C. The medium contained potato 200 g/L, sucrose 20 g/L, crude sea salt 20 g/L. After 7 days of fermentation, the cultures were centrifuged at 5000 rpm at 4°C to isolate the mycelia and the ferment broths. The mycelia were extracted with 100 mL of methanol overnight and the broths were extracted with 150 mL of ethyl acetate in total three times to yield the methanol extract and the ethyl acetate extract, respectively, then the two extracts of each strain were filtered, combined, and evaporated to dryness *in vacuo* by rotary evaporator. The resulting crude extract was finally dissolved in 2.5 mL of methanol for assay. In a clean air bench, 50 μL of extract was added using a pipette to a sterile paper disc (6 mm diameter, Whatman), which was air dried and placed on the surface of the medium seeded with test organisms [41]. Likewise, the plates were incubated and measured for inhibition zones. The average of three repeated paper disks was taken to evaluate the activity. The broad spectrum antibacterial agent ampicillin (dose: 60 μg/mL) and antifungal agent nystatin (dose: 200 μg/mL) were taken as positive control and methanol was used as negative and blank control.

### 4.4. Morphological Taxonomy

Isolate DLEP2008014 was inoculated onto PDA Petri dishes and incubated for 5 days at 25°C in darkness to observe the colonies’ morphology and measured their diameters. A filtrate paper tape with a square hole in center was wetted, placed on a glass slide and sterilized. Then a few mycelia were inoculated on the margin of the square hole of the paper tape and incubated together with a piece of wet filtrate paper in a Petri dish for two days. The morphology of the conidia chains was observed under a Nikon 80i upright microscope, and then the filtrate paper tape was removed and the morphology of the conidia observed by the usual glass slide specimen method. This isolate was also cultivated on 1/4 PCA (potato carrot agar) Petri dishes for 7 days at 25°C under natural illumination for colonial morphological survey [17].

Isolate DLEN2008016 was cultivated on MEA plates for 7 days at 25°C in darkness to observe the colonies’ morphology and measured their diameters. A cellulose tape was used to attach the conidiophores and conidia from the colonial margins. Then put the cellulose tape on the glass slide with the inoculated side upwards, added a drop of lactophenol cotton blue stain, and observed their morphology by usual glass slide specimen method [20].

Isolate DLEP2008005 was cultivated on PDA plates for 5 days at 25°C in darkness to measure the colonies’ diameters, and was continuously incubated for 3 days under natural illumination to observe the colonies’ morphology. A square piece of culture medium was cut together with mycelia, put on a sterile glass slide, and covered with a coverslip, and the the slide incubated in the Petri dish for a week. The coverslip was transferred to a new slide to make a usual glass slide specimen for observation [21].

### 4.5. Molecular Taxonomy

The fungal DNA isolation was performed using the Universal Genomic DNA Extraction Kit DV811A (Takara, Dalian, P.R. China) according to the manufacturer’s protocol. The procedure included cell lysis, digestion of RNA by RNase A, removing of precipitates and cell debris, DNA shearing, DNA precipitation and purification. The DNA was obtained dissolved in 50 μL elution buffer supplied by the manufacturer.

PCR was then performed using TaKaRa Ex Taq polymerase (Takara, Dalian, P.R. China) and the primer pair ITS1 and ITS4 in an Takara Thermal cycler Dice TP600 due to the following protocol: 1) initial denaturation 94°C/5 min; 2) denaturation 94°C/0.5 min; 3) annealing 55°C/0.5 min; 4) extension 72°C/1 min; 5) final extension 72°C/5 min; steps 2) – 4) repeated 30 times [42]. Then the PCR product mixture was analyzed by DNA electrophoresis on agarose gel, purified using TaKaRa Agarose Gel DNA Purification Kit DV805A, and sequenced by an ABI PRISMTM 3730XL sequencer (Takara, Dalian, P.R. China) with the primer Seq Forward (5′-GAGCGGATAACAATTTCACACAGG-3′). The sequence data had been submitted to and deposited at GenBank (accession numbers: FJ618520 to FJ618522).

The ITS1-5.8 S-ITS2 rDNA sequences (463–515 bp) were used to search the GenBank database with the BlastN 2.2.19+ algorithm to reveal closest matches to the ITS1-5.8 S-ITS2 rDNA sequences for known species. Sequences were aligned with representative fungal ITS1-5.8 S-ITS2 rDNA sequences using Clustal X version 1.81 and a phylogenetic tree was constructed using the Molecular Evolutionary Genetics Analysis (MEGA) software version 4.0. Bootstrap values were calculated using 1,000 bootstrap samples, and neighbor-joining was used to infer tree topologies. The phylogenetic tree was rooted with *Peziza depressa*.

## Figures and Tables

**Figure 4:**
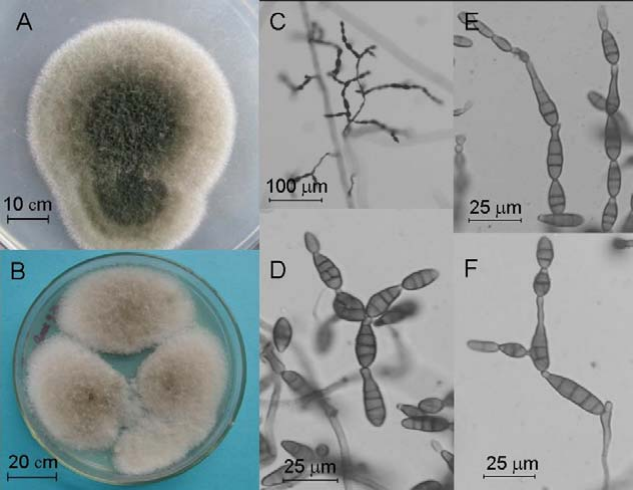
Colonies of culture DLEP2008014 grown on 1/4 PCA for seven days under natural illumination (A), colonies on PDA for five days in darkness (B), image of conidia chains cultured on filtrate paper for two days (C), image of conidia cultured on filtrate paper for two days (D–F).

**Figure 5:**
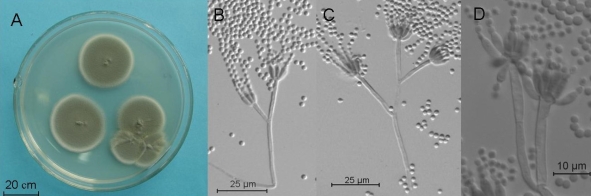
Colonies of culture DLEN2008016 grown on MEA for seven days (A), image of conidia and conidiophores from a seven-day MEA culture (B–D).

**Figure 6:**
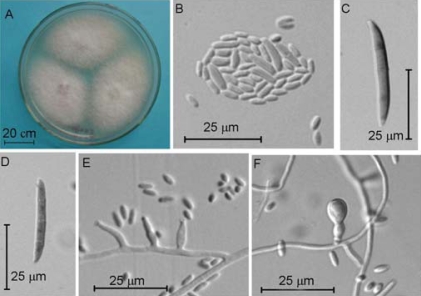
Colonies of isolate DLEP2008005 on PDA for 7 days in darkness (A), image of microconidia (B), image of macroconidia (C–D), image of microconidia generated on the top of phialides (E), and image of chlamydospores (F).

**Table 1:** The result of sample collection and the epi- and endophytic fungal isolation.

origin	type	Strain number	origin	type	Strain number
**Seaweeds**			*Ulva pertusa*	epi-	DLEP2008012
Rhodophyta				endo-	DLEN2008015
*Rhodomela confervoides*	epi-	DLS2008004		endo-	DLEN2008023
	epi-	DLS2008005	**Higher plants**		
	endo-	DLEN2008024	Magnoliophyta		
*Gelidium amansii*	epi-	DLS2008006	*Zostera marina*	epi-	DLEP2008008
	endo-	DLEN2008021		epi-	DLEP2008020
*Gracilaria lemaneiformis*	epi-	DLEP2008010	**Invertebrates**		
	epi-	DLEP2008011	Mollusca		
	epi-	DLEP2008016	*Mopalia retifera*	epi-	DLS2008001
	endo-	DLEN2008003		epi-	DLS2008002
	endo-	DLEN2008005		epi-	DLS2008003
*Ahnfeltiopsis flabelliformis*	epi-	DLEP2008001	Spongia		
	epi-	DLEP2008004	*Hymeniacidon perlevis*	epi-	DLEP2008005
	epi-	DLEP2008017		epi-	DLEP2008013
Phaeophyta				endo-	DLEN2008006
*Sargassum thunbergii*	epi-	DLEP2008014		endo-	DLEN2008009
	epi-	DLEP2008015		endo-	DLEN2008010
	endo-	DLEN2008002		endo-	DLEN2008014
	endo-	DLEN2008004		endo-	DLEN2008017
	endo-	DLEN2008011		endo-	DLEN2008018
	endo-	DLEN2008016		endo-	DLEN2008019
*Colpomenia sinuosa*	epi-	DLEP2008018		endo-	DLEN2008022
Chlorophyta			Coelenterata		
*Enteromorpha prolifera*	epi-	DLEP2008002	*Anthopleura stell*	epi-	DLEP2008009
	endo-	DLEN2008012		epi-	DLEP2008019
	endo-	DLEN2008020			
